# p53 controls hepatitis C virus non-structural protein 5A-mediated downregulation of GADD45α expression via the NF-κB and PI3K–Akt pathways

**DOI:** 10.1099/vir.0.046052-0

**Published:** 2013-02

**Authors:** Du Cheng, Lei Zhao, Leiliang Zhang, Yongfang Jiang, Yi Tian, Xinqiang Xiao, Guozhong Gong

**Affiliations:** 1 Liver Diseases Center, Department of Infectious Diseases, Second Xiangya Hospital, Xiangya Medical School, Central South University, Changsha 410011, PR China; 2 Department of Infectious Diseases, Union Hospital, Tongji Medical College, Huazhong University of Science and Technology, Wuhan 430022, PR China; 3 MOH Key Laboratory of Systems Biology of Pathogens, Institute of Pathogen Biology, Chinese Academy of Medical Sciences & Peking Union Medical College, Beijing 100176, PR China

## Abstract

Growth arrest and DNA-damage-inducible gene 45-α (GADD45α) protein has been shown to be a tumour suppressor and is implicated in cell-cycle arrest and suppression of cell growth. The hepatitis C virus (HCV) non-structural 5A (NS5A) protein plays an important role in cell survival and is linked to the development of hepatocellular carcinoma (HCC). However, the role of HCV NS5A in the development of HCC remains to be clarified. This study sought to determine whether GADD45α mediates HCV NS5A-induced cellular survival and to elucidate the molecular mechanism of GADD45α expression regulated by HCV NS5A. It was found that HCV NS5A downregulated GADD45α expression at the transcriptional level by decreasing promoter activity, mRNA transcription and protein levels. Knockdown of p53 resulted in a similar decrease in GADD45α expression to that caused by HCV NS5A, whilst overexpression of p53 reversed the HCV NS5A-mediated downregulation of GADD45α. HCV NS5A repressed p53 expression, which was followed by a subsequent decrease in GADD45α expression. Further evidence was provided showing that HCV NS5A led to increases of phosphorylated nuclear factor-κB and Akt levels. Inhibition of these pathways using pharmacological inhibitors or specific small interfering RNAs rescued HCV NS5A-mediated downregulation of p53 and GADD45α. It was also found that HCV NS5A protein and depletion of GADD45α increased cell growth, whereas ectopic expression of GADD45α eliminated HCV NS5A-induced cell proliferation. These results indicated that HCV NS5A downregulates GADD45α expression and subsequently triggers cellular proliferation. These findings provide new insights suggesting that HCV NS5A could contribute to the occurrence of HCV-related HCC.

## Introduction

Chronic infection with hepatitis C virus (HCV) is a major contributor to the high and rising incidence of hepatocellular carcinoma (HCC) worldwide (Chen & Morgan, 2006). Despite the successful development of the HCV subgenomic replication and infectious JFH1 (Japanese fulminant hepatitis 1) virus model, the mechanisms underlying HCV-mediated tumorigenesis are still not fully understood (Wakita *et al.*, 2005). HCV, a member of the family *Flaviviridae*, is a single-stranded, positive-sense RNA genome of ~9.5 kb (Choo *et al.*, 1989). The HCV genome transcript produces a large polyprotein that is cleaved proteolytically to form ten mature proteins (C, E1, E2, p7, NS2, NS3, NS4A, NS4B, NS5A and NS5B; Lindenbach & Rice, 2005). Among these viral proteins, non-structural protein 5A (NS5A) has received extensive attention. HCV NS5A is a pleiotropic phosphoprotein. The effects of NS5A on interferon signalling, as well as on the regulation of cell growth and apoptosis, have been highlighted over the past decade, demonstrating that this protein is indeed of critical importance for HCV (Macdonald & Harris, 2004). Accumulating experimental evidence suggests that HCV NS5A interacts with and affects the activity of a number of cellular kinases, including modulation of the phosphoinositol-3-kinase (PI3K)–Akt cell-survival pathway (Street *et al.*, 2004), suppression of apoptosis through either a p53-dependent or -independent mechanism (Lan *et al.*, 2002; Peng *et al.*, 2010) and perturbation of cell growth and differentiation through cell-cycle control genes (Ghosh *et al.*, 1999). We reported previously that the HCV NS5A protein alters intracellular calcium levels, induces oxidative stress and activates nuclear factor (NF)-κB (Gong *et al.*, 2001). Additionally, NS5A modulates HCV replication as a component of the replication complex (Shirota *et al.*, 2002). However, the detailed mechanisms by which NS5A leads to cell survival remain unclear.

Growth arrest and DNA-damage-inducible gene 45α (GADD45α) was originally identified *in vitro* by subtractive hybridization screening in UV-irradiated Chinese hamster cells (Fornace *et al.*, 1988). Human GADD45α is located on the short arm of chromosome 1 at 1p31.1–31.2 (Hollander *et al.*, 1993). Although GADD45α is a p53 effector gene, p53-independent induction may also be achieved (Fornace *et al.*, 1989). Additional studies have demonstrated the ability of GADD45α to interact with cellular kinases, including Akt, c-Jun N-terminal kinase and p38 mitogen-activated protein kinase (Hughes *et al.*, 2009; Jin *et al.*, 2003; Tront *et al.*, 2010). Consequently, GADD45α is involved in the regulation of cell proliferation, DNA repair, the cell cycle and apoptosis (Siafakas & Richardson, 2009). Regulation of the cell cycle and cell growth is integral for cell survival. Initial functional studies with GADD45α revealed that overexpression of GADD45α led to growth suppression in numerous cell types, primarily through activation of the G2 checkpoint in the cell cycle. When GADD45α is deleted or repressed, cells show uncontrolled proliferation (Zhan *et al.*, 1994). Furthermore, NF-κB-mediated cell survival has been shown to be dependent on suppression of GADD45α expression (Zerbini *et al.*, 2004). However, the regulation and biological function of GADD45α are complex and have not been fully elucidated.

Previous reports have found that GADD45α is downregulated in HCV-positive cirrhotic HCC patients (Gramantieri *et al.*, 2005), but direct evidence linking a loss or gain of GADD45α function in HCV-mediated liver diseases is lacking. Further studies are necessary to understand more precisely the mechanisms by which HCV infections alter GADD45α expression in the liver and the pathological outcomes of GADD45α dysfunction in HCV infection.

In the present study, we investigated the biological significance and regulatory mechanism of GADD45α expression in response to HCV NS5A-induced cell survival in human hepatoma cells. Here, we report that p53 acts as a mediator in downregulation of the GADD45α gene by HCV NS5A via cooperation of the NF-κB and PI3K–Akt pathways. The resulting reduction in GADD45α triggers cell survival.

## Results

### HCV NS5A downregulates GADD45α expression

**Fig. 1.**
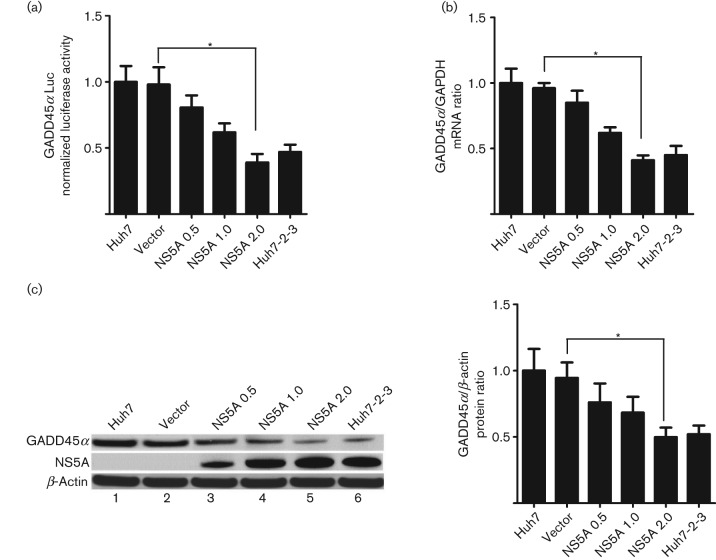
HCV NS5A downregulates GADD45α promoter activity, mRNA transcription and protein expression. (a) A GADD45α promoter–luciferase (Luc) reporter plasmid (1.0 µg) was co-transfected with the indicated plasmids at the indicated doses (µg) into Huh7 cells for 48 h and a luciferase assay was performed. (b) GADD45A mRNA levels were analysed by quantitative PCR. GADD45α mRNA levels were normalized to glyceraldehyde-3-phosphate dehydrogenase (GAPDH) levels to calculate the GADD45/GAPDH ratio, with the control non-transfected Huh7 cells having a ratio of 1. (c) Representative Western blot for GADD45α, NS5A and β-actin expression (left). Quantification of the GADD45α protein levels in Huh7-2-3 cells and in Huh7 cells transfected with NS5A-expressing vector or empty vector (right). Results are shown as means±sem of three independent experiments. *P<0.05.

A previous study has shown that GADD45α is downregulated in HCC patients (Gramantieri *et al.*, 2005). Thus, we initially investigated whether HCV NS5A could regulate GADD45α expression in hepatocytes. A GADD45α promoter reporter vector was constructed and a luciferase assay was conducted. We found that GADD45α promoter activity in NS5A-expressing Huh7 (NS5A-Huh7) cells was significantly lower than that in either Huh7 or vector-expressing Huh7 (vector-Huh7) cells. This downregulation was dose dependent (0.39-fold compared with the non-transfected control Huh7 cells at 2.0 µg NS5A, *P*<0.05; Fig. 1a). To investigate further whether the downregulation of GADD45α was consistent in the HCV subgenomic replicon system, we assessed GADD45α promoter activity in Huh7-2-3 cells, a Huh7-derived cell line stably harbouring the full-length self-replicating HCV subgenomic RNA (genotype 1b). More physiologically, much lower GADD45α promoter activity was also observed in Huh7-2-3 cells compared with Huh7 and vector-Huh7 cells (~0.47-fold compared with the control Huh7 cells, *P*<0.05; Fig. 1a). GADD45α mRNA transcript and protein levels were detected using quantitative PCR and Western blotting, respectively. Consistently, GADD45α mRNA transcript (0.41-fold at 2.0 µg NS5A, *P*<0.05; Fig. 1b) and protein levels (Fig. 1c) were steadily reduced in NS5A-Huh7 and Huh7-2-3 cells compared with Huh7 and vector-Huh7 cells. The expression levels of NS5A protein in NS5A-Huh7 and Huh7-2-3 cells were confirmed by Western blotting (Fig. 1c, left panel, lanes 3–6). These data demonstrated that HCV NS5A represses GADD45α expression by decreasing its promoter activity, mRNA transcription and protein levels *in vitro*.

### HCV NS5A-induced downregulation of GADD45α expression is p53 dependent

**Fig. 2.**
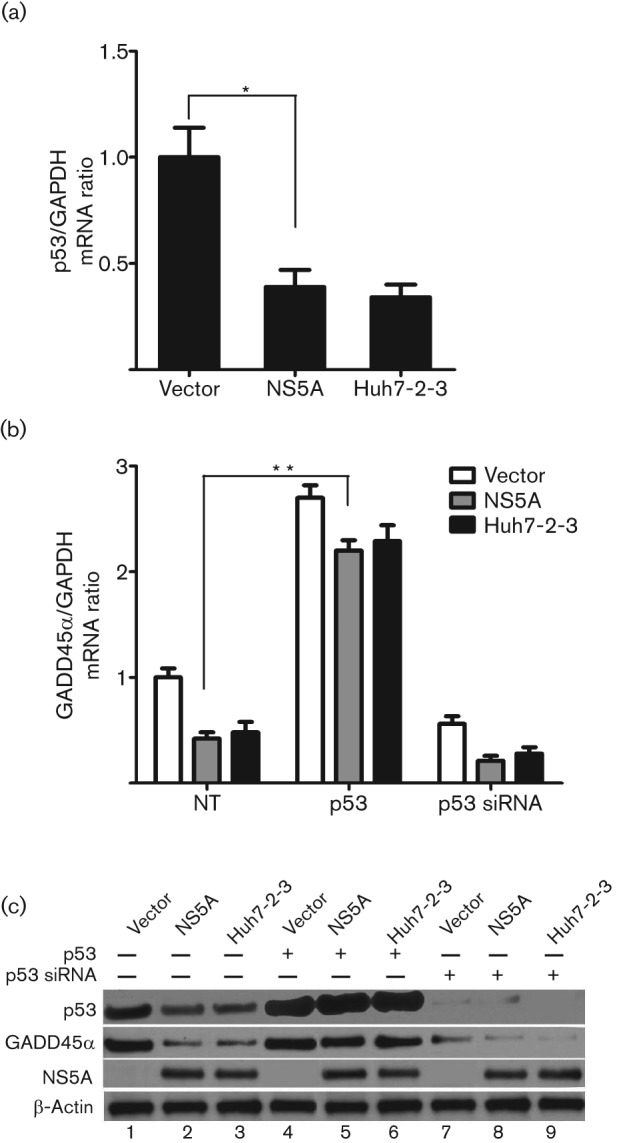
p53 mediates HCV NS5A-induced downregulation of GADD45α expression. Stable vector-Huh7, NS5A-Huh7 and Huh7-2-3 cells were seeded into a 24-well plate 24 h before transfection with a p53 plasmid (0.5 µg) or p53 siRNA (50 nM) for 72 h. (a) Quantitative PCR for p53 mRNA levels. *P<0.05. (b) Quantitative PCR for GADD45α mRNA expression in the presence of p53 overexpression (p53) or knockdown (p53 siRNA). **P<0.001. Results for (a) and (b) are shown as means±sem of three independent experiments. (c) Representative Western blotting for p53, GADD45α, NS5A and β-actin protein levels. p53 protein was knocked down by p53 siRNAs (lanes 7–9). NS5A protein was detected in NS5A-Huh7 (lanes 2, 5 and 8) and Huh7-2-3 cells (lanes 3, 6 and 9).

We next asked how HCV NS5A regulates GADD45α expression. Previous studies have shown that GADD45α expression is mediated by both p53-dependent and -independent mechanisms (Chen *et al.*, 2001; Schumm *et al.*, 2006). We reported previously that HCV NS5A eliminates p53 protein function by interfering with p53–DNA binding (Gong *et al.*, 2004). Thus, we explored whether the downregulation of GADD45α expression by HCV NS5A was linked to p53 expression. First, we observed that p53 mRNA transcription (0.34–39-fold compared with the vector control, *P*<0.05; Fig. 2a) and p53 protein levels (Fig. 2c, lanes 2–3) were significantly decreased in stable NS5A-Huh7 and Huh7-2-3 cells compared with stable vector-Huh7 cells.

Next, to explore whether p53 was involved in NS5A-mediated downregulation of GADD45α, we examined GADD45α expression in stable NS5A-Huh7 and Huh7-2-3 cells in the presence of p53 overexpression or knockdown using small interfering RNAs (siRNAs). As shown in Fig. 2(b), overexpression of p53 significantly increased GADD45α mRNA levels in vector-Huh7 (2.70- vs 1.0-fold, *P*<0.001), NS5A-Huh7 (2.20- vs 0.42-fold, *P*<0.001) and Huh7-2-3 cells (2.29- vs 0.48-fold, *P*<0.001), whereas p53 knockdown decreased GADD45α mRNA transcription in vector-Huh7 (0.56- vs 1.0-fold, *P*<0.05), NS5A-Huh7 (0.21- vs 0.42-fold, *P*<0.05) and Huh7-2-3 cells (0.28- vs 0.48-fold, *P*<0.05), compared with the corresponding untreated groups. These data indicated that GADD45α expression was p53 dependent. GADD45α protein levels in the presence of p53 overexpression were significantly higher than those in the presence of p53 knockdown (concomitantly or not with NS5A and HCV replication; Fig. 2c, lanes 4–6 vs lanes 7–9).

Taken together, these data support the view that HCV NS5A downregulates p53 expression, which is followed by a subsequent reduction in GADD45α expression.

### HCV NS5A-induced downregulation of p53 and GADD45α expression is mediated by NF-κB and PI3K–Akt pathways

**Fig. 3.**
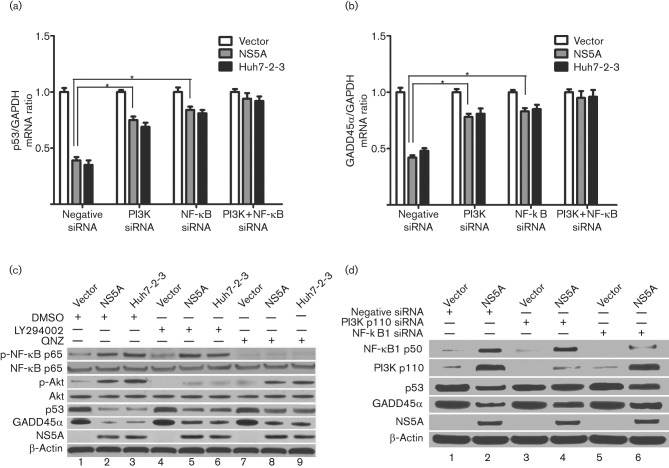
The combination of inhibition of PI3K and NF-κB further rescues p53 and GADD45α expression. Stable vector-Huh7, NS5A-Huh7 and Huh7-2-3 cells were seeded into a 24-well plate with PI3K and NF-κB siRNAs (50 nM), as indicated. The cells were incubated for 72 h before harvest. (a, b) Effect of siRNAs targeting PI3K and NF-κB on p53 (a) and GADD45 (b) mRNA levels, determined by quantitative PCR. Results are shown as means±sem of three independent experiments. *P<0.05. (c) Effect of NF-κB activation inhibitor QNZ and PI3K inhibitor LY294002 on the phosphorylation of NF-κB and Akt and on the protein levels of p53 and GADD45α, determined by Western blotting. Cells were incubated with QNZ (5 µM) and LY294002 (50 µM) for 12 h before harvest. (d) Effect of siRNAs targeting PI3K and NF-κB1 on the protein levels of p53 and GADD45α determined by Western blotting. The expression levels of NF-κB1 and PI3K protein were knocked down by the respective siRNAs.

NF-κB and PI3K–Akt cellular kinases are important signalling pathways affecting p53 expression and functions (Boehme *et al.*, 2008; Royds *et al.*, 1998). Therefore, we assessed the effect of these kinases on the expression of p53 and GADD45α (Fig. 3). Western blotting revealed that the phosphorylation of NF-κB and Akt was much higher in NS5A-Huh7 and Huh7-2-3 cells compared with the low basal levels of phosphorylation in vector-Huh7 cells (Fig. 3c, lanes 2 and 3). The NF-κB activation inhibitor 6-amino-4-(4-phenoxyphenylethylamino) quinazoline (QNZ) and PI3K inhibitor LY294002 were used to block NF-κB and PI3K–Akt pathways, respectively. Accordingly, QNZ and LY294002 eliminated the phosphorylation of NF-κB (Fig. 3c, lanes 8 and 9) and Akt (Fig. 3c, lanes 5 and 6) in NS5A-Huh7 and Huh7-2-3 cells, respectively. Moreover, QNZ and LY294002 partially rescued p53 and GADD45α protein expression compared with the control DMSO in NS5A-Huh7 (Fig. 3c, lanes 8 and 5) and Huh7-2-3 cells (Fig. 3c, lanes 9 and 6), respectively.

To determine further the specific effect of the PI3K–Akt and NF-κB pathways on p53 and GADD45α expression, we used siRNAs to knockdown these kinases. We found that the downregulation of p53 mRNA transcription was partially rescued by PI3K (increased to 0.75-fold) and NF-κB siRNAs (increased to 0.84-fold) compared with the negative-control siRNAs (0.39-fold, *P*<0.05; Fig. 3a) in NS5A-Huh7 cells. The combination of NF-κB and PI3K siRNAs cooperatively rescued p53 mRNA transcription to a higher level than that with individual NF-κB or PI3K siRNAs (increased to 0.92–94-fold, *P*<0.05; Fig. 3a). NF-κB and PI3K siRNAs exhibited a similar rescue effect on p53 mRNA transcription in Huh7-2-3 cells to that observed in NS5A-Huh7 cells (Fig. 3a). Consistently, GADD45α mRNA levels with the combination of NF-κB and PI3K siRNAs were much higher than those with either NF-κB or PI3K siRNA alone (~0.95- vs 0.78–85-fold, *P*<0.05; Fig. 3b) in NS5A-Huh7 and Huh7-2-3 cells. Western blotting confirmed that the expression levels of PI3K and NF-κB protein were knocked down by PI3K and NF-κB siRNAs, respectively (Fig. 3d, lanes 4 and 6). PI3K and NF-κB siRNAs also partially rescued p53 and GADD45α protein levels compared with the negative-control siRNAs in NS5A-Huh7 cells, which confirmed our previous data (Fig. 3d, lanes 4 and 6).

Overall, these data suggested that HCV NS5A stimulates the activation of the NF-κB and PI3K–Akt pathways, which cooperatively downregulate p53 expression. Decreased p53 subsequently leads to the repression of GADD45α.

### GADD45α mediates HCV NS5A-induced cell proliferation

**Fig. 4.**
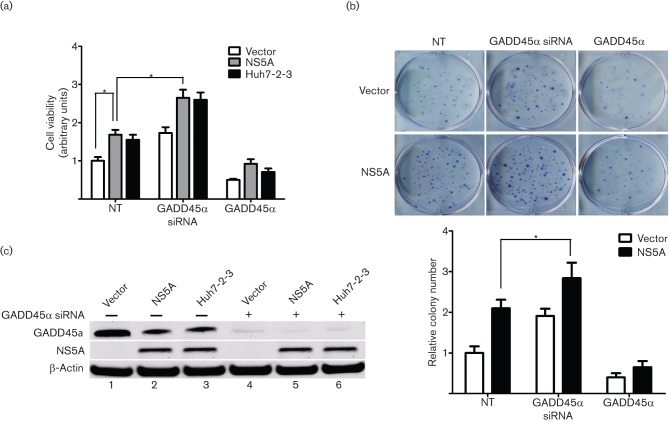
Role of GADD45α in HCV NS5A-induced cell proliferation. (a) Stable vector-Huh7, NS5A-Huh7 and Huh7-2-3 cells were seeded into a 96-well plate 24 h before transfection with GADD45α siRNA (50 nM) or GADD45α plasmid (0.5 µg), as indicated. The cells were incubated for 72 h before the cell viability assay. Results are shown as means±sem of three independent experiments. *P<0.05. (b) The effect of GADD45α on cellular growth was further confirmed by a colony formation assay. The upper panel shows representative images of colony formation in stable vector-Huh7 and NS5A-Huh7 cells transfected with GADD45α plasmid or GADD45α siRNAs. Quantitative analysis of colony numbers is shown in the lower panel (means±sem of three independent experiments). (c) Western blotting confirmed that the GADD45α protein was knocked down by GADD45α siRNAs. The NS5A protein was detected in stable NS5A-Huh7 and Huh7-2-3 cells.

A number of studies have suggested that HCV NS5A promotes cell survival (Ghosh *et al.*, 1999; Majumder *et al.*, 2001; Street *et al.*, 2004). It has been indicated that depletion or downregulation of GADD45α induces uncontrolled cell proliferation (Siafakas & Richardson, 2009). We therefore speculated that the downregulation of GADD45α accounted for the HCV NS5A-induced cell survival. First, we assessed the effect of HCV NS5A on cell proliferation in human hepatoma cells. HCV NS5A induced a significant increase in cell viability compared with the vector control (1.68-fold, *P*<0.05; Fig. 4a). Consistently, Huh7-2-3 cells exhibited a similar induction effect on cell viability to that observed in NS5A-Huh7 cells (1.55-fold, *P*<0.05; Fig. 4a). To investigate further whether the increased cellular proliferation in stable NS5A-Huh7 and Huh7-2-3 cells was associated with GADD45α expression, GADD45α siRNAs or GADD45-expressing vector were transfected into these cells. As shown in Fig. 4(a), knockdown of GADD45α significantly increased cell viability in vector-Huh7 (1.73- vs 1.0-fold, *P*<0.05), NS5A-Huh7 (2.65- vs 1.68-fold, *P*<0.05) and Huh7-2-3 (2.60- vs 1.55-fold, *P*<0.05) cells compared with the corresponding untreated groups, indicating that GADD45α is one of the pivotal genes in the pathways of cell proliferation. Furthermore, remarkably, overexpression of GADD45α eliminated the increase in cell viability in NS5A-Huh7 (0.92- vs 1.68-fold, *P*<0.05) and Huh7-2-3 (0.71- vs 1.55-fold, *P*<0.05; Fig. 4a) cells compared with the corresponding untreated groups, once again suggesting that GADD45α at least partially has a significant role in cell proliferation induced by NS5A and HCV replication. The induction effect of NS5A on cell growth was confirmed further using a colony formation assay. The numbers of colonies formed in NS5A-Huh7 cells were much higher than those in vector-Huh7 cells (increased to 1.89–2.31-fold compared with the vector control, *P*<0.001; Fig. 4b), which confirmed our previous data that HCV NS5A induces cell proliferation. Moreover, colonies formed in GADD45α siRNA-transfected cells were significantly more numerous and larger in size than those formed in GADD45α plasmid-transfected cells (concomitantly or not with NS5A; Fig. 4b), further implicating that a decrease in or depletion of GADD45α triggers cell proliferation. Western blotting confirmed the expression of HCV NS5A in stable NS5A-Huh7 and Huh7-2-3 cells. GADD45α protein was knocked down by the GADD45α siRNAs (Fig. 4c, lanes 4–6).

Taken together, these results are in agreement with our hypothesis that HCV NS5A downregulates GADD45α expression, which in turn contributes to cell survival.

## Discussion

Although many studies have focused on the carcinogenesis of HCV, the detailed mechanism has not been fully elucidated. The HCV NS5A protein is a critical component of HCV because of its ability to modulate the host-cell interferon response and multiple cellular pathways (Macdonald & Harris, 2004). Previous studies have shown that GADD45α expression is downregulated in HCC patients (Gramantieri *et al.*, 2005). Here, we have provided direct evidence that HCV NS5A downregulates GADD45α gene expression via a p53-dependent mechanism, which in turn leads to cellular proliferation of human hepatoma cells *in vitro*.

The expression of GADD45α is known to be regulated by both p53-dependent (after exposure to ionizing radiation) and p53-independent (after UV radiation) pathways depending on the type of genotoxic stress (Hughes *et al.*, 2009). In our study, overexpression of p53 ameliorated the downregulation of GADD45α induced by HCV NS5A. In contrast, knockdown of p53 further enhanced GADD45α downregulation (Fig. 2). This study indicated that p53 acts as a mediator and leads to GADD45α decrease. Thus, p53 appears to be at the head of the key cellular pathways, and the association of p53 with NS5A may disrupt the normal cell cycle and growth.

PI3K is a heterodimeric enzyme consisting of a p85 regulatory subunit and a p110 catalytic subunit (Cantley, 2002). Our data suggested that NS5A upregulated the catalytic subunit p110 (Fig. 3d), which could provide new evidence that NS5A stimulates PI3K activity. However, the mechanisms for the upregulation of catalytic subunit p110 by NS5A are only partially understood. Previous reports have shown that p85 inhibits the catalytic activity of p110. NS5A binds to the SH3 domain of the p85 regulatory subunit, which in turn relieves the p85-mediated inhibition of the p110 catalytic subunit and activates lipid kinase activity (Street *et al.*, 2004, 2005). Moreover, NS5A can activate PI3K through N-Ras (Mannová & Beretta, 2005). Further experiments are needed to explore whether NS5A can activate PI3K indirectly through other cellular kinases, or inflammatory or stress stimuli.

NF-κB proteins in mammalian cells include NF-κB1 (p50/p105), NF-κB2 (p52/p100), RelA (p65), RelB and c-Rel. p105 is proteolytically processed by the proteasome to produce p50. The most predominant form of NF-κB is the p50/p65 heterodimer (Liu & Malik, 2006; Siebenlist *et al.*, 1994). Here, we presented evidence that NS5A activated the phosphorylation of p65 (Fig. 3c) and upregulated p50 (Fig. 3d). We have shown previously that NS5A induces oxidative stress, leading to the activation of NF-κB (Gong *et al.*, 2001). We speculated that, following oxidative stress, p105 might be subjected to phosphorylation, ubiquitination and proteasomal degradation processes, resulting in the increasing release of p50 (Karin & Ben-Neriah, 2000).

It has been shown that NF-κB and Akt negatively regulate p53 expression (Wu & Lozano, 1994; Yamaguchi *et al.*, 2001). Previous reports have shown that upregulation of NF-κB represses GADD45α expression, which is essential for cells to escape from programmed cell death (Gupta *et al.*, 2006). The Akt inhibitor has also been found to induce GADD45α expression and cell-cycle arrest at the G2/M check point, which in turn leads to cell apoptosis (Zhu *et al.*, 2008). This study also indicated that HCV NS5A induced activation of the NF-κB and PI3K–Akt pathways. Using siRNAs for these kinases, NF-κB and PI3K siRNAs partially rescued the downregulation of p53 and GADD45α expression (Fig. 3). We showed that HCV NS5A activated the NF-κB and PI3K–Akt pathways, which in turn repressed p53 and GADD45α expression. This finding highlights a new role for these kinase pathways in the control of cell growth initiated by HCV NS5A.

GADD45α is involved in maintenance of genomic stability, DNA repair and suppression of cell growth (Siafakas & Richardson, 2009). GADD45α induces cell-cycle G2/M arrest (Mullan *et al.*, 2001) and depletion of GADD45α leads to uncontrolled cell growth (Siafakas & Richardson, 2009). Knockdown of GADD45α markedly accelerated HCV NS5A-induced cell proliferation, and overexpression of GADD45α reversed HCV NS5A-induced proliferation in human hepatoma cells compared with the untreated NS5A group (Fig. 4). These data indicate the significant relevance of NS5A protein in HCV-mediated pathogenesis and a contributory role of GADD45α in cell proliferation. More comprehensive HCC cell lines or non-neoplastic hepatocyte lines might be necessary to understand better the effect of HCV NS5A on hepatocyte survival.

**Fig. 5.**
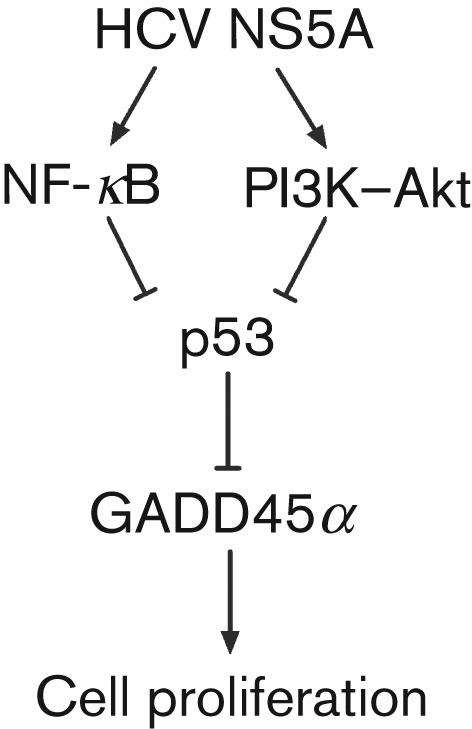
Proposed mechanism for the downregulation of GADD45α expression by HCV NS5A, contributing to cell survival in human HCC cells. The NF-κB and PI3K–Akt pathways cooperatively influence HCV NS5A-mediated downregulation of p53 and subsequently inhibit GADD45α expression. The resulting GADD45α reduction contributes to cell proliferation.

Previous studies have shown that HCV NS5A promotes cell proliferation by interacting with the PI3K–Akt cell survival pathway (Street *et al.*, 2004). NS5A may also interact directly with the cell-cycle regulatory gene p21/waf1, resulting in increased cell proliferation and a transformed phenotype (Majumder *et al.*, 2001). In addition, NS5A could disrupt the apoptotic process through a protein kinase R- or p53-dependent mechanism (Gale *et al.*, 1999; Lan *et al.*, 2002). In our study, we propose a unique model in which HCV NS5A induces the phosphorylation of NF-κB and Akt, which in turn cooperatively repress p53 expression (Fig. 5). The decreased p53 acts as a mediator leading to the downregulation of GADD45α expression, which ultimately triggers cell proliferation. However, we cannot exclude the possibility that NS5A also interacts with other kinases or cell-cycle control genes, resulting in cellular survival.

A previous report has shown that full-length HCV J6/JFH1 induces apoptosis in Huh7.5 cells (Deng *et al.*, 2008). These results seem to be inconsistent with our data that HCV NS5A promotes cellular survival. The discrepancy could be a reflection of inherent differences in host response to acute versus chronic viral infection. The J6/JFH1-infected Huh7.5 cells model is an acute model. We speculate that J6/JFH1, under acute infection conditions, provokes a host immune response for viral clearance, which thus results in portal and lobular inflammatory infiltration, apoptosis and necrosis. Once a chronic infection becomes established, HCV can utilize various strategies (for example, NS5A downregulates cell-cycle control genes) to inhibit cellular apoptosis and promote cell proliferation, which is beneficial for viral survival. A previous study reported that the virus replication, viral titre and measurements of levels of cell apoptosis all decreased with an increase in HCV infection time, providing evidence in support of our speculation (Zekri *et al.*, 2011). Moreover, a recent study has shown that chronic HCV infection in chimpanzees leads to reduced susceptibility to cytokine-induced apoptosis (Saeed *et al.*, 2011). Another study has shown that HCV induces the activation of transcription factor Nrf2, contributing to a cellular survival pathway in JFH1 cells, which is in agreement with our findings (Burdette *et al.*, 2010).

In conclusion, the current study provides new evidence that downregulation of GADD45α is a critical event leading to HCV NS5A-induced cell proliferation in hepatocytes. These data also elucidate the contributory role of NS5A in HCV-related HCC. Further molecular, clinical and epidemiological studies are now warranted to determine the detailed mechanisms by which HCV NS5A alters the function of GADD45α and the role of GADD45α alterations in the pathogenesis of HCV in the liver. Furthermore, it remains to be established whether the GADD45α gene represents a therapeutic target for preventing the progression of HCV-related HCC.

## Methods

### 

#### Cell lines and culture.

Human HCC Huh7 cells (obtained from the Institute of Cell Biology, Shanghai, China) were grown in Dulbecco’s modified Eagle’s medium (DMEM; Cellgro) supplemented with 10 % FBS (Gibco) and 1 % penicillin/streptomycin (Gibco). Huh7-2-3 cells (harbouring a full-length HCV 1b replicon) were grown in 10 % FBS/DMEM supplemented with 400 µg G418 (Cellgro) ml^−1^. The cells were maintained in a humidified incubator at 37 °C with 5 % CO_2_.

#### Transient DNA transfections and construction of a stable NS5A-expressing cell line.

Cells were transfected with plasmids using Lipofectamine 2000 (Invitrogen) according to the manufacturer’s instructions. Briefly, Huh7 cells (4×10^5^ per well) in a six-well plate were transfected using 5 µl Lipofectamine 2000 and 2 µg total DNA. The transfection mixture was removed after 6 h and substituted with complete medium. To establish a stable NS5A-expressing cell line, Huh7 cells were seeded in 60 mm dishes and transfected with pRc-NS5A (HCV 1b) or pRc/CMV empty vector plasmid. The cells were allowed to recover for 48 h before the addition of 800 µg G418 ml^−1^ to the culture medium for initial selection of stable colonies. G418-resistant colonies were maintained in medium supplemented with 400 µg G418 ml^−1^. Colonies of G418-resistant NS5A-expressing (NS5A-Huh7) and vector-expressing (vector-Huh7) cells were confirmed by PCR, Western blotting and sequencing. The GADD45α plasmid was purchased from OriGene.

#### Reagents.

Cells were incubated with NF-κB activation inhibitor QNZ (5 µM) and PI3K inhibitor LY294002 (50 µM) (both from EMD Chemicals) for 12 h. The inhibitor stock solution was dissolved in 1 % DMSO, which was used alone as a negative control.

#### siRNAs and transfection.

The indicated siRNAs (Dharmacon) and negative-control siRNAs (Cell Signalling) were transfected at 50 nM final concentration into cells using HiPerFect Transfection Reagent (Qiagen) according to the manufacturer’s instructions. The siRNAs used for gene knockdown were as follows: siGENOME SMARTpool GADD45α siRNAs, siGENOME SMARTpool PI3K siRNAs, ON-TARGETplus SMARTpool NF-κB1 siRNAs and ON-TARGETplus SMARTpool TP53 siRNAs (Thermo Scientific). The protein expression of each gene following knockdown was confirmed by Western blotting.

#### Construction of GADD45α promoter reporter plasmid and luciferase assay.

The GADD45α promoter reporter plasmid was cloned by PCR from human genomic DNA using the primers 5′-AGGTACCAGGCTCTCTGTGGAAGGTAACGA-3′ (sense) and 5′-AAGATCTGGGCTCCTCCTCCTGTGCC-3′ (antisense). The sequences were designed based on the published sequence of GADD45α (GenBank accession no. L24498.1; Hollander *et al.*, 1993; Zhan *et al.*, 1998). The resultant 1097 bp fragment, which contained the human GADD45α gene sequences spanning the region between −952 and +145 bp, was inserted into the *Kpn*I and *Bgl*II sites of a pGL3-Basic Luciferase Reporter Vector (Promega). A fixed level of GADD45–luciferase (1.0 µg) was co-transfected with the indicated plasmids at various doses into Huh7 and Huh7-2-3 cells for 48 h. All assays were corrected for the total amount of protein using a Bio-Rad protein assay. Firefly luciferase assays were then conducted using a Luciferase Reporter Assay System (Promega).

#### Quantitative PCR.

Total cellular RNA was isolated using RNeasy Mini columns (Qiagen) and reverse transcribed by random priming with a High Capacity cDNA Reverse Transcription kit (Applied Biosystems). The RNA was quantified by real-time PCR using a DyNAmo HS SYBR Green qPCR kit (Finnzyme). Human GAPDH was used as a control for basal RNA levels. The primers used for GADD45α were 5′-GAGAGCAGAAGACCGAAAGGA-3′ (sense) and 5′-CAGTGATCGTGCGCTGACT-3′ (antisense); and for p53 were 5′-GAGGTTGGCTCTGACTGTACC-3′ (sense) and 5′-TCCGTCCCAGTAGATTACCAC-3′ (antisense).

#### Western blotting.

Cells were lysed using RIPA buffer containing 1 % NP-40, 0.1 % SDS, 10 mM Tris/HCl (pH 7.4), 1 mM EDTA, 150 mM NaCl and protease inhibitor cocktail, and subsequently sonicated. The proteins were separated by SDS-PAGE with NuPAGE Novex pre-cast 4–12 % Bis-Tris gradient gels (Invitrogen) and transferred to PVDF membranes. The primary antibodies used were as follows: rabbit anti-actin (Sigma), rabbit anti-GADD45α, rabbit anti-phospho-Akt (Ser473) and anti-phospho-NF-κB p65 (Ser536), rabbit anti-unphosphorylated Akt and NF-κB p65, rabbit anti-PI3 kinase p110 and anti-NF-κB1 p105/p50 (Cell Signalling) and rabbit anti-NS5A (ViroGen). The secondary antibody was HRP-conjugated ECL donkey anti-rabbit IgG (Amersham Biosciences). An ECL Western Blotting Detection kit (Amersham) was used to detect the chemiluminescent signals.

#### Cell viability assay.

Cell viability was assessed using a Cell Titre-Glo Luminescent Cell Viability Assay kit (Promega) according to the manufacturer’s protocol. Cells were seeded at a density of 10^4^ cells per well (in 100 µl DMEM with 10 % FBS) in 96-well white plates for 72 h. Cell Titre-Glo viability reagent (100 µl) was then added to each well. The plates were mixed for 2 min on an orbital shaker and incubated at room temperature in the dark for 10 min. Luminescence was measured using an FLX800 microplate reader (BioTek).

#### Colony formation assay.

A colony formation assay was performed using monolayer cultures. Stable vector-Huh7 and NS5A-Huh7 cells (4×10^5^ per well) were seeded in a six-well plate and transfected with GADD45α siRNAs or GADD45α-expressing plasmid. After 48 h of transfection, the cells were collected and seeded (300 cells per well) in a fresh six-well plate. After incubation for 14 days, colonies (≥50 cells per colony) were counted after staining with 1 % crystal violet solution (Sigma). All experiments were performed in triplicate wells three times.

#### Data analysis.

Data analysis was performed using a two-tailed Student’s *t*-test with pooled variance. Data are expressed as means±sd of at least four sample replicates, unless stated otherwise. A level of *P*<0.05 was considered significant.
